# Supplementary figures and images for: Unexpected distribution of the 4-formylaminooxyvinylglycine (FVG) biosynthetic pathway in Pseudomonas and beyond
Source: PLoS One. 2021 Apr 23;16(4):e0247348. doi: 10.1371/journal.pone.0247348 (PMC8064604; doi:10.1371/journal.pone.0247348)

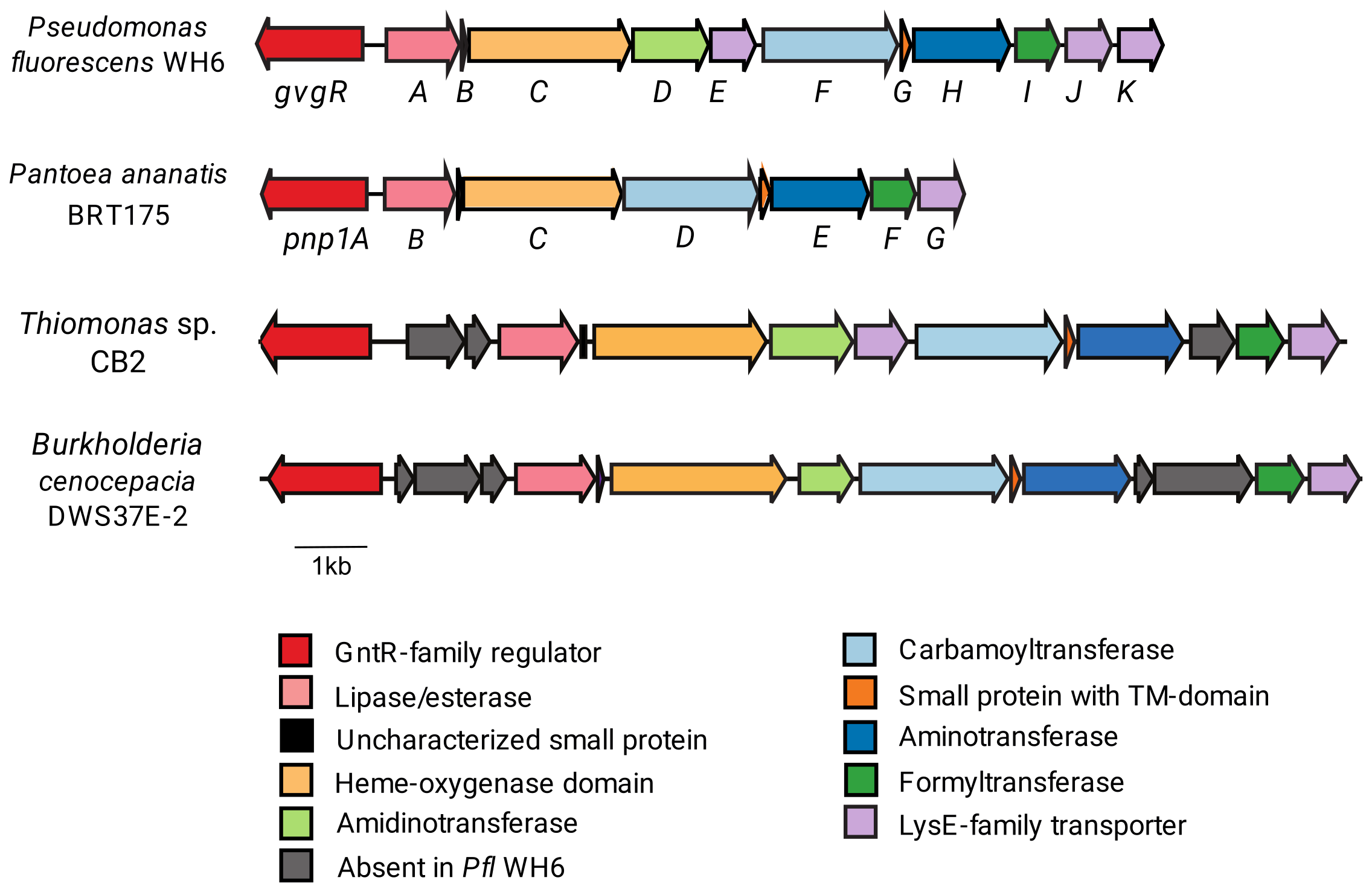

Supplement: S1 Fig — Comparison of gvg gene clusters in Pseudomonas fluorescens WH6, Pantoea ananatis BRT175, Thiomonas sp. CB2 and Burkholderia cenocepacia. Gene arrows are colored by function and are to scale. Gray arrows indicate genes not present in gvg clusters from P. fluorescens. (TIF) [file pone.0247348.s001.tif]

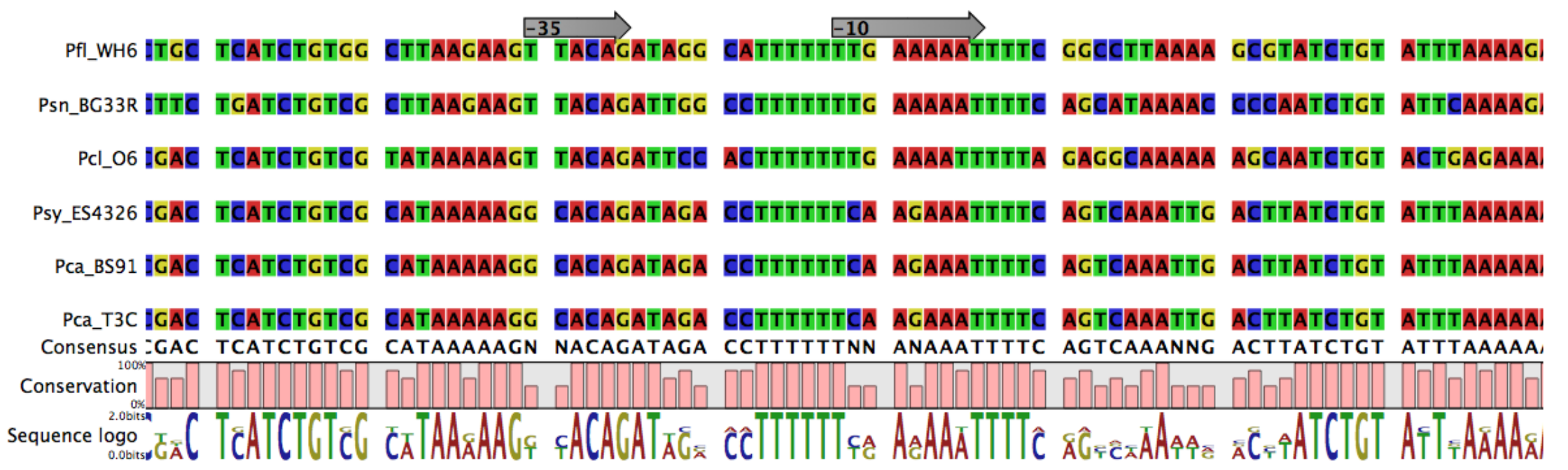

Supplement: S2 Fig — Comparison of the promoters in the intergenic region between gvgR and gvgA of the gvg clusters from Pseudomonas fluorescens, P. chlororaphis and P. syringae strains. The locations of the -35 and -10 promoter sites for P. fluorescens WH6 are indicated by gray arrows above the sequences. (TIF) [file pone.0247348.s002.tif]

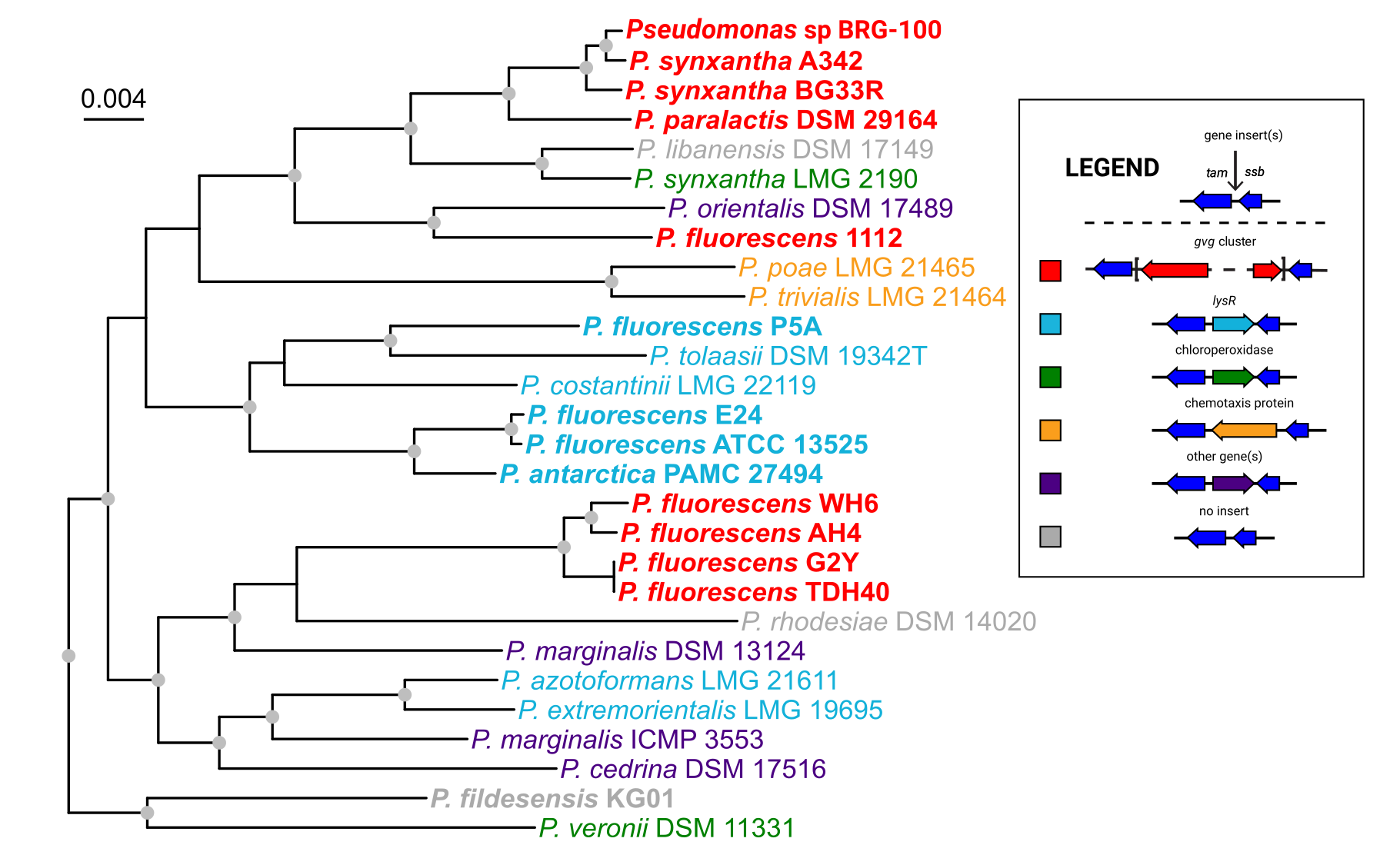

Supplement: S3 Fig — This is a subset of Fig 2 focusing on the Pseudomonas fluorescens subgroup. The putative identity of genes present between the tam and ssb genes in a given strain are indicated by color (red = gvg locus; turquoise = lysR protein; green = chloroperoxidase protein; orange = chemotaxis protein; purple = other genes; gray = no insert). Strains containing a gvg locus are shown in bold. (TIF) [file pone.0247348.s003.tif]
